# Informed Choice for Participation in Down Syndrome Screening: Development and Content of a Web-Based Decision Aid

**DOI:** 10.2196/resprot.4291

**Published:** 2015-09-21

**Authors:** Mette Maria Skjøth, Helle Ploug Hansen, Eva Draborg, Claus Duedal Pedersen, Ronald F Lamont, Jan Stener Jørgensen

**Affiliations:** ^1^ Research Unit of Gynecology and Obstetrics Department of Gynecology and Obstetrics, Institute of Clinical Research Odense University Hospital, University of Southern Denmark Odense Denmark; ^2^ Centre for Innovative Medical Technology Odense University Hospital Odense Denmark; ^3^ Department of Public Health Research Unit of General Practice University of Southern Denmark Odense Denmark; ^4^ Department of Public Health, Health Economics University of Southern Denmark Odense Denmark; ^5^ Division of Surgery Northwick Park Institute for Medical Research Campus University College London London United Kingdom

**Keywords:** decision support intervention, decision making, informed choice, prenatal diagnosis, pregnancy, development, Web-based intervention, eHealth tool, telemedicine

## Abstract

**Background:**

In Denmark, all pregnant women are offered screening in early pregnancy to estimate the risk of having a fetus with Down syndrome. Pregnant women participating in the screening program should be provided with information and support to allow them to make an informed choice. There is increasing interest in the use of Web-based technology to provide information and digital solutions for the delivery of health care.

**Objective:**

The aim of this study was to develop an eHealth tool that contained accurate and relevant information to allow pregnant women to make an informed choice about whether to accept or reject participation in screening for Down syndrome.

**Methods:**

The development of the eHealth tool involved the cooperation of researchers, technology experts, clinicians, and users. The underlying theoretical framework was based on participatory design, the International Patient Decision Aid Standards (IPDAS) Collaboration guide to develop a patient decision aid, and the roadmap for developing eHealth technologies from the Center for eHealth Research and Disease Management (CeHRes). The methods employed were a systematic literature search, focus group interviews with 3 care providers and 14 pregnant women, and 2 weeks of field observations. A qualitative descriptive approach was used in this study.

**Results:**

Relevant themes from pregnant women and care providers with respect to information about Down syndrome screening were identified. Based on formalized processes for developing patient decision aids and eHealth technologies, an interactive website containing information about Down syndrome, methods of screening, and consequences of the test was developed. The intervention was based on user requests and needs, and reflected the current hospital practice and national guidelines.

**Conclusions:**

This paper describes the development and content of an interactive website to support pregnant women in making informed choices about Down syndrome screening. To develop the website, we used a well-structured process based on scientific evidence and involved pregnant women, care providers, and technology experts as stakeholders. To our knowledge, there has been no research on the combination of IPDAS standards and the CeHRes roadmap to develop an eHealth tool to target information about screening for Down syndrome.

## Introduction

Interest in the use of information and communication technology in health care is increasing. Interventions based on eHealth have increased, but there is a lack of empirical evidence about the benefits, and greater awareness with respect to the development process, implementation, and evaluation is needed [1]. In obstetrics, digital solutions have been shown to be a useful form of health care delivery, but there is a need for further validation and evaluation [2].

Prenatal screening for Down syndrome (a genetic condition caused by the presence of an extra copy of chromosome 21) [3] is well established. In Denmark, all pregnant women are offered such screening in the first trimester, determined using a combination of maternal age, sonographic measurement of fetal nuchal translucency, and maternal serum concentrations of free beta-human chorionic gonadotropin and pregnancy-associated plasma protein-A. This screening offers a detection rate of approximately 90% for a false positive rate of 5% [4]. Pregnant women with a fetus considered to be at increased risk of Down syndrome are offered further invasive diagnostic tests such as chorionic villous sampling or amniocentesis, which carry a procedure-related risk of spontaneous miscarriage of about 0.5% to 1% [5]. The main purpose of screening for Down syndrome is to assist pregnant women in choosing whether to accept or reject participation based on informed choice [6,7]. Informed choice relies on information of a certain quality and reflection of patient values and is based on the principle that it is unethical for patients not to be informed of the consequences of health care interventions and an informed choice is associated with a better outcome [8]. Pregnant women need information about the condition for which the test is offered, the method by which the test is carried out, the consequences of the test results, and the fact that the test is optional [9]. Women of advanced maternal age tend to choose invasive tests because to their age and not their individual risk assessment [10]. Several studies have demonstrated the benefits of making an informed choice compared with an uninformed choice [11-15], yet not all pregnant women do so [16-18]. Accordingly, it is important to focus on ways to inform pregnant women about such options.

Pamphlets, audiotapes, workbooks, and videotapes are examples of interventions that prepare patients to decide on health care options [19]. Such aids have been shown to improve knowledge and support informed choice [20]. Care providers should be aware that patients use the Internet to search for information on health-related issues and should support this use [21]. In 2012 in Denmark, 99% of couples with children had access to a personal computer at home, and 86% of families had access to the Internet [22]. Accordingly, a large proportion of the Danish population has access to health-related websites which are among the most frequently used [23]. Web-based health information is unregulated and varies in quality and consistency [24]. Web-based interventions and eHealth technologies can change behavior, improve knowledge, and deliver health care information in a more flexible and time-efficient manner but this requires focus on development and implementation [25-27]. Patients are using the Internet in several ways with respect to health information: searching for health information, participating in support groups, and consulting with health professionals [28,29]. A Swedish study demonstrated that pregnant women often use the Internet to find information on topics related to pregnancy and concluded that antenatal care providers should be able to guide pregnant women to high-quality, Web-based information [30]. The aim of this study was to develop an eHealth tool that contained accurate and relevant information to improve pregnant women’s ability to make an informed choice about whether to accept or reject participation in screening for Down syndrome. Elements of participatory design, an approach for developing technical solutions to real-world problems in cooperation with stakeholders, were used to define problems and create sustainable solutions [31].

## Methods

### Theoretical Framework

#### International Patient Decision Aid Standards Collaboration Guide

The IPDAS Collaboration is an international group of researchers, practitioners, and stakeholders that developed a checklist of approved criteria to ensure the quality of patient decision aids. The IPDAS guide was used to ensure the quality of the content and fulfill the aim of developing a useful and effective tool. The criteria are grouped into three main areas (content, development, and effectiveness) and consist of 12 quality dimensions (Textbox 1) [32].

**Quality dimensions outlined by the International Patient Decision Aid Standards (IPDAS) Collaboration.**

Twelve quality dimensions:

- Using a systematic development process
- Providing information about options
- Presenting probabilities
- Clarifying and expression values
- Using patient stories
- Guiding or coaching in deliberation and communication
- Disclosing conflicts of interest
- Delivering patient decision aids via the Internet
- Balancing the presentation of options
- Using plain language
- Basing information on up-to-date scientific evidence
- Establishing effectiveness

#### Center for eHealth Research and Disease Management Roadmap

**Figure 1:**
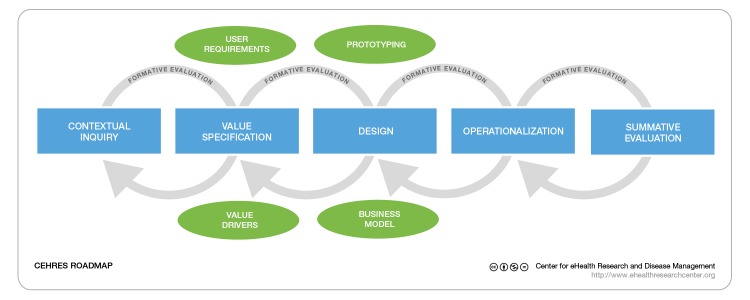
CeHRes roadmap.

The CeHRes roadmap was designed to guide planning, coordination, and execution of the developmental process of eHealth technologies [27,33]. The roadmap was based on a holistic approach whereby individual elements in a complex system are interrelated and influence each other. The roadmap was used as guidance for the development of the eHealth tool to ensure a continuing process in accordance with the approach of participatory design. According to the roadmap, the development of an eHealth technology starts with multidisciplinary project management and undergoes 5 main steps: contextual inquiry, value specification, design, operationalization, and summative evaluation (Figure 1) [27,34]. Our study applied elements of participatory design, an approach for developing technical solutions to real-world problems in close cooperation with stakeholders and end users, to define problems and create sustainable solutions for practice [31].

### Steps 1 and 2: Contextual Inquiry and Value Specification

#### The Process of Development of the eHealth Tool

In this phase the project management and the approach of the system design were clarified to ensure an effective process. A research group was established to run the development process of the intervention. The first author (MMS) was the project manager responsible for the process, content, and design. As part of their higher education qualification project, three multimedia design students from Lillebaelt Academy of Professional Higher Education were engaged to design and build the intervention. To ensure a clinical approach, experts in maternal fetal medicine provided advice [27]. The research group, developers, and expert group worked in collaboration to develop the intervention, and pregnant women were regularly involved in the process [35].

#### Background Literature

The databases PubMed and Embase were searched systematically for studies that investigated the effects of interventions compared to conventional care in pregnant women considering Down syndrome screening and generated the basis for a systematic review. The review was used as background information for this study and has been published elsewhere [36].

#### Design

A qualitatively descriptive method was used to assess pregnant women’s needs for information about Down syndrome screening. Qualitative description is a useful method to obtain knowledge about an individual’s experience in an area that is poorly understood and a target for an intervention [37-39]. The method was used throughout the study from data collection to analysis. The qualitatively descriptive method can help to focus on the experiences of the pregnant women and stay very close to the data obtained [37]. The goal of the method is descriptive and uses low inference interpretation to present findings in everyday language. Data collection in qualitatively descriptive studies is often directed at the *who*, *what*, and *where* of experiences and usually includes minimally to moderately structured open-ended individual and/or focus group interviews [39].

#### Setting

The study was performed at the Maternal Fetal Medicine Clinic at Odense University Hospital. About 4100 pregnant women are referred to the hospital every year, and the clinic performs about 18,000 ultrasound scans per year. In Denmark, Down syndrome screening incurs no cost to the pregnant women.

#### Data Collection

One interview with three care providers was held in the early stage of the study. The care providers were recruited from the Maternal Fetal Medicine Clinic at Odense University Hospital. Inclusion criterion was professional experience in maternal fetal medicine. Three care providers with different professional backgrounds were recruited: a consultant, a nurse, and a midwife. All three care providers were women with more than five years of experience in maternal fetal medicine. The interview with the care providers was established to elucidate the current clinical pathway for Down syndrome screening, the staff’s perception of the pathway, and new ideas on how to inform pregnant women. While three care providers might be considered a relatively small number for a focus group interview, this provided the opportunity for in-depth questions and greater involvement of the participants.

Two focus group interviews with pregnant women who had formerly participated in Down syndrome screening at Odense University Hospital were held to identify the perceived information that was required, the source of the information, and the challenges with respect to Down syndrome screening. One interview with eight pregnant women was held in the early stage of the study, and one interview with six pregnant women was held in the middle of the study. The pregnant women were recruited from midwife consultations at the time of nuchal translucency scanning at Odense University Hospital. The inclusion criteria were: healthy (physically and mentally) pregnant women with uncomplicated pregnancies who spoke and understood Danish. All participants were informed about the study and gave their informed content to participate. The women varied in age, parity, and education to ensure wide representation. The pregnant women’s ages ranged from 21 to 39 years, and they were expecting their first, second or third child. Pregnant women in the first interview were included during the whole period of pregnancy, whereas women in the second interview were included during first or second trimester of pregnancy.

All focus group interviews were moderated by MMS and one other interviewer, and a semi-structured guide was used to elicit the participants’ experiences. An interview guide, based on the literature search and field observations (only the third interview) was used to keep the conversation focused on the following themes: knowledge about Down syndrome screening, challenges in connection with the screening process, and possible improvements. All three interviews were digitally recorded.

Supplementary to the qualitative interviews, field observation was carried out to authenticate the challenges of providing information about Down syndrome screening. Field observations comprised direct observation in the Maternal Fetal Medicine Clinic at Odense University Hospital and were held after the two first interviews. Over a period of two weeks, MMS and one of the technical experts observed pregnant women, their partners, and care providers in the clinic. The participants were observed and interviewed at these consultations and at information meetings in the clinic. Their responses were noted by both observers. This method can help to overcome the discrepancy between what participants say and what they do and also may help to uncover behavior which the participants are not aware of [40]. In this study, field observations were carried out to qualify the meaningfulness and understanding of the first two interviews in order to prepare for the third interview (content and interview guide) rather than generating individual data.

#### Data Analysis

The qualitatively descriptive approach was also used for data analysis. All three focus group interviews were digitally recorded. The interviews were transcribed verbatim by a secretary to optimize the analysis. First, the transcribed material was read to obtain an overview and impression of the data. Furthermore, recurrent themes were identified. Second, the data were read again and relevant text sections were made. The data were coded into meaningful titles according to the themes identified in the first step. Third, all text sections with similar codes were categorized into general themes. The data were reread to reflect and identify common factors and differences. Finally, to ensure correct coding and categorization, the data were reviewed for coherence and reallocated if discrepancies were found [37,41]. Coding and categorization was done by hand and in Microsoft Word. Due to the relatively small amount of data, computer analysis programs were not required.

Field observations were carried out to qualify the meaningfulness and understanding of the first two interviews to prepare for the third interview (content and interview guide) rather that generating individual data. Hence, the notes from the field observations were used to support the findings of the first two interviews and to prepare for the last interview and was not analyzed separately.

### Steps 3 and 4: Design and Operationalization

These two phases refer to the design of prototypes that fit with the values and user requirements and concern the practical development and employment of the technology. Based on the themes identified, an interactive website with information about Down syndrome screening was developed. The website reflected accurately the process and information provided by the Maternal Fetal Medicine Clinic at Odense University Hospital in line with national and international standards. The website was developed with a focus on seven elements (Table 1).

**Table 1:** Important elements in the development of the eHealth tool.

Element	Action
Goals	Goals were established to ensure a clear direction for the development of the intervention. The goals were to ensure that knowledge was imparted to pregnant women about Down syndrome screening, to support them in making an informed choice, to present existing and new information in new ways to the pregnant women, to reflect a professional and friendly service from the hospital, and to provide all information with a neutral attitude with respect to the different options available to the pregnant women.
Design	The design of the website was based on user values and was simple in design, look, font, and colors. Other websites, both national and international, were searched for inspiration. The website was developed in WordPress, a content management system for setting up websites, and Google Analytics was used for user information and search engine optimization. Images used on the website were selected or created in cooperation with the expert group to ensure medical accuracy and realism.
Mock-up	A mock-up model of the website was used through the development process to oversee the direction of the work.
Clinical content	The clinical content of the website was based on national and international guidelines for Down syndrome screening, user requirements of pregnant women, and input and reviews from the expert group. The clinical content also took into consideration the needs of pregnant women to have informed choice about Down syndrome screening, the method of testing, interpretation of negative or positive results and the fact that the test was optional [9,42]. Furthermore, the eHealth tool allowed pregnant women to become actively engaged in the decision-making process [43].
Language	To ensure plain language, an expert in health communication reviewed and revised the text before it was used on the website.
Decisional conflict	Screening for Down syndrome was characterized by a decisional conflict with no single best choice. The eHealth tool aim was to provide information about the condition, the options, benefits and harms, probabilities, and interpretation [20].
Communication risks	Making an informed choice about Down syndrome screening involves dealing with risks and statistical issues. In accordance with recommendations for risk communication, the Web-based interventions were designed with special focus on presenting risk in alternative ways using graphics, plain language, and consideration of how statistics are presented [44]. Presenting information in frequency format is beneficial to convey statistical information [45].

### Step 5: Evaluation

Finally, the user-friendliness of the eHealth tool was tested. The prototype version of the intervention was tested on six pregnant women and two experts who used the website and gave feedback and suggested improvements. The website was also evaluated using the IPDAS checklist for developing patient decision aids.

## Results

### Steps 1 and 2: Contextual Inquiry and Value Specification

#### Themes Identified by Care Providers

The professionals concluded that some of the pregnant women had based their choice on a predetermined decision rather than an informed choice. Faced with an increased risk of Down syndrome, the pregnant women were frustrated and had difficulty making a choice because of lack of knowledge. Furthermore, the professionals discovered that pregnant women found it difficult to deal with the meaning and significance of cut-off values. The importance of having enough information to make an informed choice was also evident. The professionals suggested developing Web-based material to supplement the existing means of providing information that would enable pregnant women to continue to enjoy their pregnancy. Examples of themes identified by the care providers are shown in Textbox 2. The themes were selected based on relevance and frequency in the interviews.

#### Themes Identified by Pregnant Women

To make an informed choice, the majority of the pregnant women in the focus group stated that they needed more information, although this varied by degree. Some women reported that they expected a normal outcome and therefore did not require much information. Others reported that they wanted to know everything about the screening. Common to all was the expectation of obtaining a picture of their unborn child at the time of scan, and this caused some consternation among care providers who felt that their role was being trivialized. Several women reported doubt with respect to the interpretation and understanding of cut-off values and sought extra information. Some of the women stated that while they received a number of booklets, they had not read them and preferred to obtain information from the Internet and from friends and family. Only a few received information from their general practitioner. Using the Internet, the pregnant women sought the experiences of other pregnant women and used a number of different sites. Several of the women stated that they liked to use websites with a chat room. They did not necessarily want to chat with others, but they liked to follow other women’s chats and to see that other women also had doubt with respect to the interpretation and understanding of cut-off values. Common to all was the need for reliable and helpful information available on a single website. Examples of themes identified by pregnant women are shown in Textbox 2. The themes were selected based on relevance and frequency in the interviews.

**Themes from interviews with pregnant women and care providers.**

Examples of themes from interviews with care providers:

- Quality of the information
- Seeking confirmation for normality
- Lack of knowledge for pregnant women at increased risk
- Different agendas between the care providers and the pregnant women
- Doubts about the meaning of the cut-off values

Examples of themes from interviews with pregnant women:

- Difficulties in making an informed choice
- Need for knowledge/information and where to find it
- No understanding of cut-off values
- Assessment of available information

### Steps 3 and 4: Design and Operationalization

Common to all pregnant women in the interviews was the need for reliable and helpful information available at a single Internet site. Based on the interviews it was decided to develop an interactive website [46] to support pregnant women in making an informed choice about Down syndrome screening. The content of the website was based upon the identified themes among care providers and pregnant women and guidelines for Down syndrome screening. The website reflected the clinical pathway at Odense University Hospital and was divided into subpages according to this (see Multimedia Appendix 1). The design of the website was based on user values and was simple in design, look, font, and colors. The majority of the topics on the website were described in written text supplemented by short videos of care providers explaining the topics and showing the screening methods. Both care providers and pregnant women reported doubt with respect to the interpretation and understanding of cut-off values. Hence, it was decided to use both static graphics and animated infographics to help visualize the text and give a better understanding of the statistical aspects of screening for Down syndrome. During the interviews with pregnant women it became clear that several of the women liked to use websites with chat forums and it was decided to include a chatroom on the website designed for pregnant women to share stories. Pictures were used on the website to reflect the present topic and to create a professional and accommodating atmosphere (see Multimedia Appendix 2). Images used on the website were selected or created in cooperation with the expert group to ensure medical accuracy and realism.

### Step 5: Evaluation

The prototype version of the intervention was tested on six pregnant women and two clinical experts who used the website and gave feedback and suggested improvements. The website was also evaluated using the IPDAS checklist for developing patient decision aids. Based on the feedback and evaluation the website was adjusted with special focus on usability.

## Discussion

### Principal Findings

This paper describes the development of an eHealth tool as an intervention to improve pregnant women’s ability to make an informed choice about Down syndrome screening. This was based on a theoretical framework to develop a patient decision aid and a roadmap to develop eHealth technologies in the form of an interactive website [46].

The development of the intervention was a complex and time-consuming process involving many people to support existing means of informing pregnant women in a more interactive manner using new information and communication techniques. Using the guide from the IPDAS standards for developing patient decision aids helped us to ensure the quality of the content and fulfill the aim of developing a useful and effective tool. The IPDAS standards are suitable for developing patient decision aid tools and have been used in the development of several patient decision-making tools [47-49]. By combining this with the CeHRes roadmap, a focused and structured collaborative developmental process was provided. The evolution of the process helped to ensure the development of an intervention that was based on the needs and wishes of pregnant women. The CeHRes roadmap has been used in several studies as guidance for the development of eHealth technologies [50,51] but to our knowledge, this is the first time that the combination of the IPDAS standards and CeHRes roadmap has been used to develop an eHealth tool specific to information about Down syndrome screening.

A qualitatively descriptive method was used to approach data. Qualitative description research closely reflects data and provides a comprehensive summary in simple language. In contrast to other qualitative research approaches, there is a sharper line between exploration and description of data [39]. Qualitative description has been criticized for being unclear and not sufficiently theory-based. However, when the approach is used for the right purpose, this criticism is unreasonable [37]. Qualitative description should be used when a description of a certain view is required, and using this method to focus on the experiences of the pregnant women has been useful for gaining information used to develop an eHealth tool. Employing a qualitatively descriptive method provided the opportunity to collaborate closely with the pregnant women in a timely and resource-effective way.

Focus group interviews helped to provide knowledge and understanding of the area of prenatal screening for Down syndrome and were an effective method in the process of developing the intervention. However, focus group interviews are less suitable for producing data on individuals, as there is less time for each individual to speak and social interaction in the group can prevent different views from being expressed. This might risk atypical views not being reported [52]. Two focus group interviews with eight and six pregnant women each were considered adequate for this study to provide background information for an intervention rather than an analysis of interaction. In addition, the topic was quite personal, which may not be ideal in a large group. Conversely, three staff members was a relatively little number for a focus group interview. However, this gave the opportunity for in-depth questions and greater involvement of the participants. Field observations provided the opportunity to authenticate the challenges of providing information about Down syndrome screening, and the pregnant women were very willing to share their thoughts and stories. It gave the opportunity to observe what questions were asked when the pregnant women had doubts and also what worked well and less well. Field observations are useful for understanding a certain phenomenon, but less so for causalities [53]; in addition, the observations are time-consuming and often unstructured. The researcher has a certain perspective and will focus on this, while other things may not be noticed. Furthermore, the researchers must be aware of their influence on the participants being observed. Working in pairs, observers can meet the challenges, share experiences, and support each other. Field observations were carried out to qualify the meaning and understanding of the first two interviews and guide the content of the third interview and were not analyzed separately. Field observations in combination with interviews were valuable in authenticating the challenges of providing information about Down syndrome screening. We gathered important data during the two weeks of field observations, and this experience should be included in future studies.

One of the strengths of our study was the involvement of technology experts, pregnant women, and care providers in the process. This resulted in the development of an intervention based on user needs that also reflected hospital practice. Patient decision aids increase knowledge and support informed choice [20]. When developing eHealth tools, it is important to prioritize the process, commit people, and invest time to make an effective and useful intervention. Goal setting affects performance and persistence and has an energizing effect [54]. All stakeholders involved in the process were familiar with the goals of the intervention and worked with common goals. The content of the website is reliable, current, and evidence-based in accordance with the recommendations of the Danish Health and Medicines Authority [6]. For sustainability, it is important to maintain and update the website. Another strength of our study is the openness of the website which can be used by all pregnant women regardless of risk. Many individuals have little understanding of statistical analysis so it is difficult for them to understand probabilities and risk [55]. We found that pregnant women had difficulty dealing with cut-off values.

### Limitations

While an expert in health communication rewrote the website and videotext, there is still a risk that not all pregnant women will cope with such information. Accordingly, it is important to consider the website as a supplement to face-to-face consultations. The website contains a chat room designed for pregnant women to share stories, which is challenging to implement. It may be possible to supplement the website with video communication. Another limitation could be the exclusion of vulnerable and non-Danish speaking participants in the study.

### Comparison With Prior Work

Other studies concerning the development of eHealth tools recognized the importance of a structured process to develop a successful intervention and used a theoretical framework for the process [48,50,56,57]. Women assigned to an interactive, computerized Prenatal Testing Decision Assisting Tool with information about prenatal testing had higher knowledge scores and less decisional uncertainty [15]. The increased use of information technology will affect health care, and more interventions in this area are likely to be seen. Pregnant women seek and care providers recommend the use of Web-based interventions to provide information. There is benefit in delivering decisions aids on the Internet, but few are developed for Web-based use [58]. Our findings indicate a need for Web-based information, and this current study shows that it is possible to develop Web-based decisions aids for patients.

### Conclusions

This paper describes the development of an interactive website [46] to inform pregnant women about screening for Down syndrome in a highly specialised obstetric unit in Denmark. The development of an interactive website to support pregnant women in making informed choices about whether to accept or reject participation in Down syndrome screening relies on a well-structured process based on scientific evidence and involving stakeholders such as pregnant women, care providers, and technology experts. The study has demonstrated how different frameworks and methods must be used in a complementary manner to develop an eHealth tool. The website supports existing forms of educating pregnant woman and is designed to support pregnant women’s ability to make an informed choice. Further research has been done to investigate whether this intervention improves pregnant women’s ability to make an informed choice with respect to screening for Down syndrome, and the results will be published at a later date.

## Appendix

Multimedia Appendix 1 Sitemap overview.

Multimedia Appendix 2 Homepage of the Graviditets Portalen website.

## Abbreviations

CeHRes: Center for eHealth Research and Disease Management
IPDAS: International Patient Decision Aid Standards
