# Supplementary material for: Informed Choice for Participation in Down Syndrome Screening: Development and Content of a Web-Based Decision Aid
Source: JMIR Res Protoc. 2015 Sep 21;4(3):e113. doi: 10.2196/resprot.4291 (PMC4704943; doi:10.2196/resprot.4291)
Supplement: Multimedia Appendix 2 [file resprot_v4i3e113_app2.pdf]

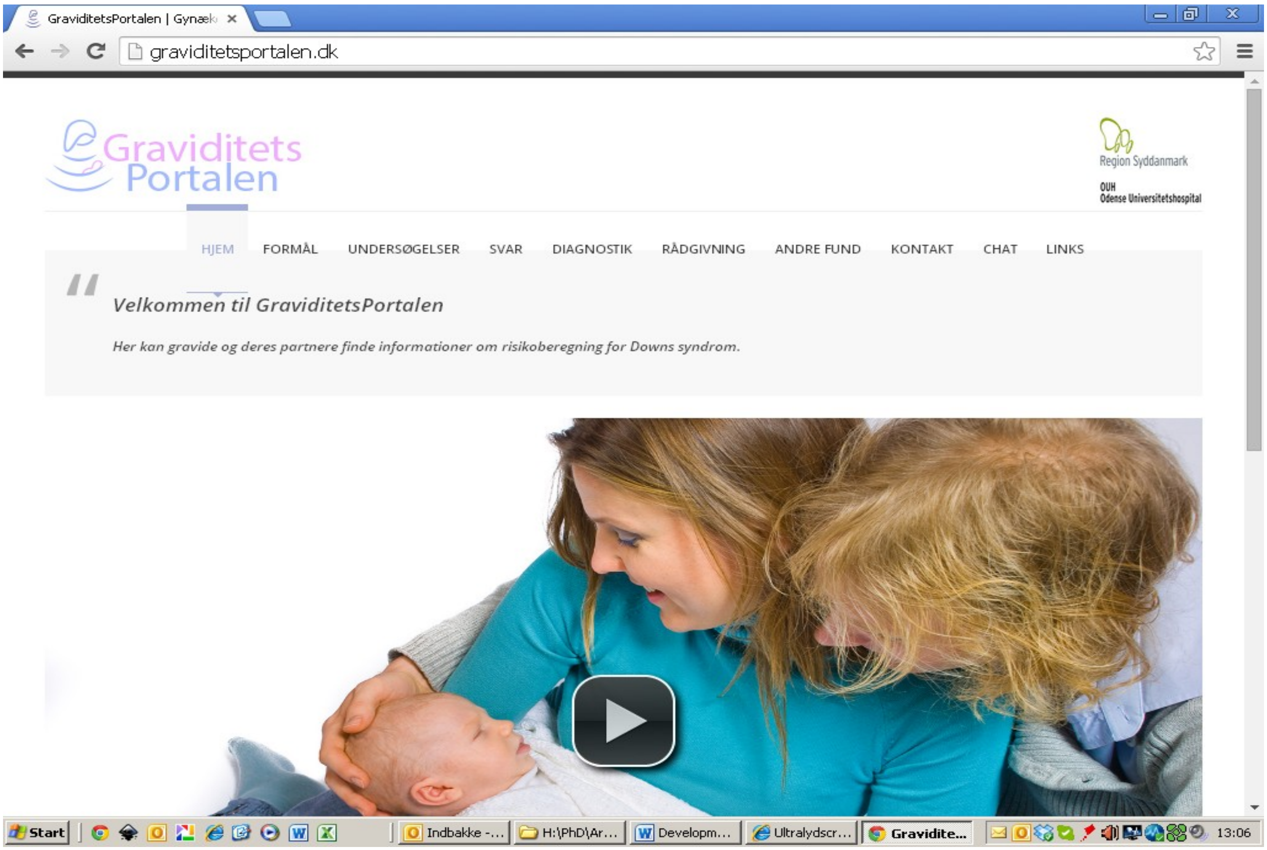
GraviditetsPortalen | Gynæk
graviditetsportalen.dk
Graviditets Portalen
Region Syddanmark
OUH
Odense Universitetshospital
HJEM
FORMÅL
UNDERSØGELSER
SVAR
DIAGNOSTIK
RÅDGIVNING
ANDRE FUND
KONTAKT
CHAT
LINKS
Velkommen til GraviditetsPortalen
Her kan gravide og deres partnere finde informationer om risikoberegning for Downs syndrom.
Start
Indbakke -...
H:\PhD\Ar...
Developm...
Ultralydscr...
Gravidite...
13:06
